# Next-generation soil monitoring: linking metagenomics, biosensors, and ecological modeling for sustainable agriculture

**DOI:** 10.3389/fmicb.2026.1861333

**Published:** 2026-07-01

**Authors:** Ricardo Romero-Arguelles, Gabriel Ruiz-Ayma, Violeta A. Rodriguez-Castro, Jose I. Gonzalez-Rojas, Mayra A. Gomez-Govea

**Affiliations:** 1 Laboratorio de Ecofisiologia, Facultad de Ciencias Biológicas, Universidad Autónoma de Nuevo León, San Nicolas de los Garza, Nuevo Leon, Mexico; 2 Laboratorio de Biológia de la Conservación y Desarrollo Sostenible, Facultad de Ciencias Biológicas, Universidad Autónoma de Nuevo León, San Nicolas de los Garza, Nuevo Leon, Mexico; 3 Laboratorio de Entomologia, Facultad de Ciencias Biológicas, Universidad Autónoma de Nuevo León, San Nicolas de los Garza, Nuevo Leon, Mexico

**Keywords:** environmental sensors, metagenomics, microbial networks, predictive modeling, soil microbiome, sustainable soil management

## Abstract

Soils represent one of the most complex and dynamic biological systems on Earth, where microbial communities play a central role in regulating ecosystem functions, including nutrient cycling, carbon sequestration, and plant productivity. However, increasing pressures from land-use intensification and climate change threaten soil health and biodiversity, highlighting the need for innovative monitoring and management approaches. In this review, we synthesize current advances in soil microbial ecology, sustainable soil management, environmental sensing technologies, and metagenomics to propose an integrative framework for soil monitoring and prediction. This review integrates environmental sensing, microbiome characterization, ecological modeling, and AI-based analytics into a unified framework for next-generation predictive soil monitoring systems. We discuss how high-resolution environmental sensors enable real-time characterization of soil physicochemical dynamics, while metagenomic approaches provide unprecedented insights into the taxonomic and functional diversity of soil microbiomes. Furthermore, we explore the role of microbial network analysis and ecological modeling in uncovering interaction patterns and predicting ecosystem responses to environmental change. The integration of these tools through machine learning and data-driven approaches is transforming soil science from a descriptive to a predictive discipline. We also address key challenges, including data standardization, scalability, and the interpretation of complex biological datasets. Finally, we highlight emerging directions such as microbiome-informed precision agriculture, microbiome engineering, and the development of soil digital twins. Together, these advances pave the way toward sustainable soil management strategies that enhance ecosystem resilience and agricultural productivity in the face of global change.

## 1 Introduction

Soil is one of the most complex and dynamic systems in the biosphere. Rather than being a mere physical substrate, it functions as a living ecosystem that harbors an extraordinary diversity of microorganisms, including bacteria, archaea, fungi, and viruses. It is estimated that soils contain a substantial fraction of global terrestrial biodiversity and play essential roles in biogeochemical cycles, climate regulation, and the maintenance of plant productivity (Bardgett and van der Putten, 2014; Fierer, 2017). Soil microorganisms drive key processes such as organic matter decomposition, nutrient mineralization, and carbon cycling, forming the functional backbone of the soil–plant–atmosphere continuum (Delgado-Baquerizo et al., 2016; Bernard et al., 2022). Accordingly, the soil microbiome has emerged as a central framework for understanding ecosystem functioning and resilience (Jansson and Hofmockel, 2020). Despite its importance, soil systems are increasingly threatened by anthropogenic pressures and rapid environmental change (Smith et al., 2016). Soil degradation—manifested through erosion, salinization, compaction, and loss of organic matter—poses a critical risk to food security and ecosystem stability (Gomiero, 2016). In parallel, climate change introduces additional stressors, including shifts in temperature and precipitation regimes, which significantly alter the structure and function of soil microbial communities (Jansson and Hofmockel, 2020; Gomiero, 2016). These changes can influence greenhouse gas fluxes, reinforcing positive climate feedbacks and reducing the soil’s capacity to act as a carbon sink (Orr et al., 2025).

**Figure 1:**
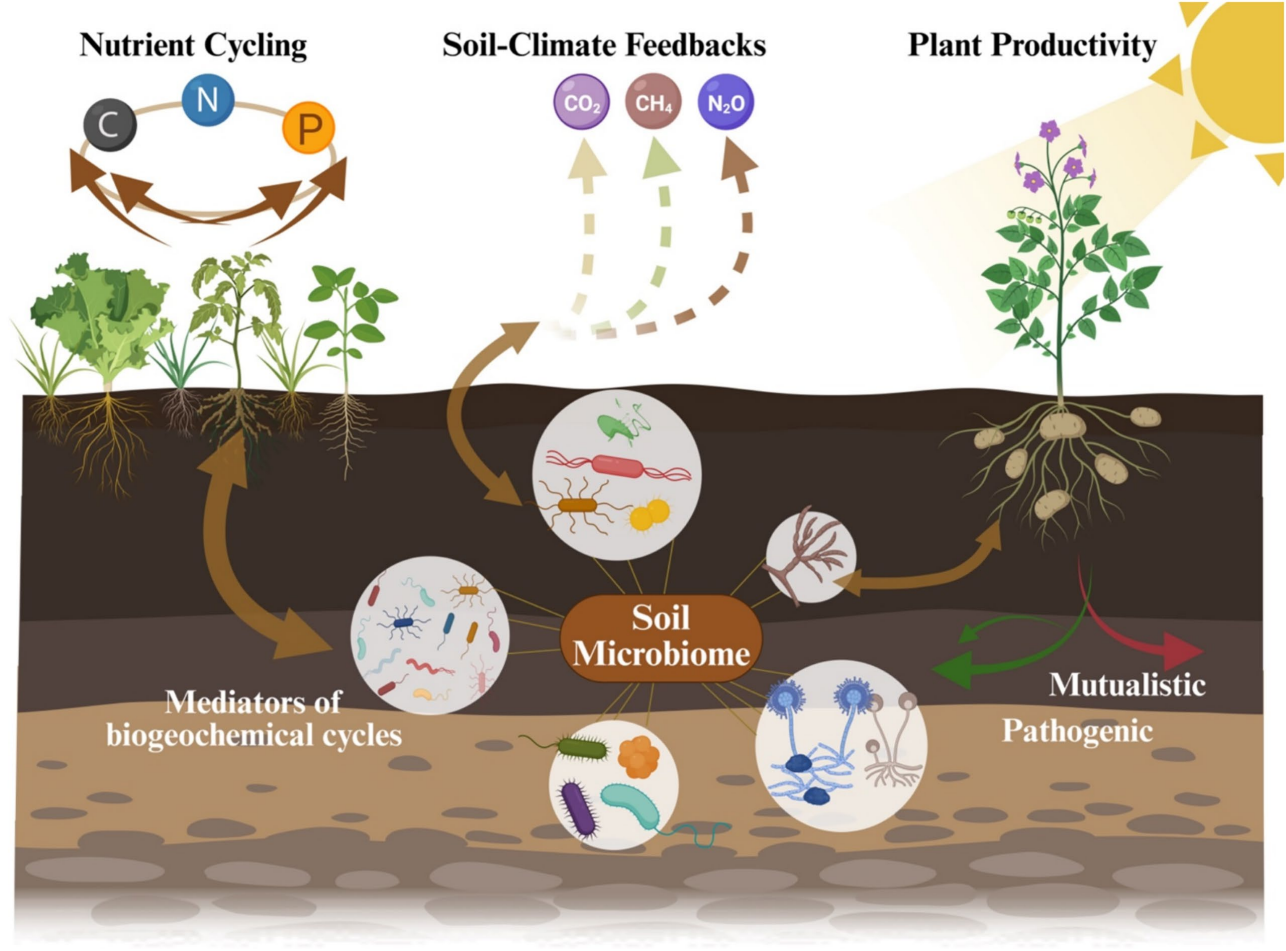
Conceptual diagram of the soil microbiome and its ecosystem functions. The figure illustrates the central role of the soil microbiome—including bacteria, archaea, and fungi—in regulating key processes such as nutrient cycling (carbon, nitrogen, and phosphorus), plant productivity, and soil–climate feedbacks. Plant–microbe interactions are depicted, encompassing both mutualistic and pathogenic relationships, along with microbial-mediated greenhouse gas fluxes (CO₂, CH₄, and N₂O). Overall, the diagram highlights how subsurface microbial networks connect biogeochemical and ecological processes and may support the stability and resilience of terrestrial ecosystems.

A major concern is the ongoing loss of soil microbial biodiversity. Recent studies suggest that reductions in microbial diversity not only alter community composition and disrupt carbon cycling processes, but also decrease carbon use efficiency and increase greenhouse gas emissions, particularly CO₂, thereby intensifying climate feedbacks associated with global warming (Bardgett and van der Putten, 2014; Delgado-Baquerizo et al., 2016; Tibbett et al., 2020; Domeignoz-Horta et al., 2020). Moreover, the simplification of microbial interaction networks can reduce ecosystem stability and resilience to disturbances, impairing processes such as nutrient cycling and plant health (Philippot et al., 2021; Delgado-Baquerizo et al., 2016). Thus, soil biodiversity loss represents both an ecological and functional challenge, with direct implications for the sustainability of agroecosystems. Addressing these challenges requires integrated approaches for soil monitoring, understanding, and management. Metagenomics has transformed soil microbiome research by enabling culture-independent taxonomic and functional profiling of microbial communities (Clagnan et al., 2024). This approach has provided key insights into plant–microbe interactions and their role in ecosystem resilience and agricultural productivity (Jansson and Hofmockel, 2020). However, metagenomics alone remains limited in linking functional potential to real-time environmental dynamics (Fierer, 2017) (Figure 1).

In parallel, advances in environmental sensing and Internet of Things (IoT) technologies have enabled real-time monitoring of soil parameters such as moisture, temperature, pH, and electrical conductivity, generating high-resolution spatial and temporal datasets (Gomiero, 2016). These tools offer new opportunities to connect microbial dynamics with environmental conditions and management practices. Nevertheless, integrating these datasets with biological information remains a key challenge. Ecological modeling provides a critical bridge to address this gap. By integrating multi-scale data, modeling approaches—including microbial network analysis, machine learning, and mechanistic simulations—allow the prediction of soil system dynamics, evaluation of management strategies, and exploration of soil–climate feedbacks (Orr et al., 2025; Philippot et al., 2021). Importantly, incorporating microbiome processes into Earth system models has been identified as a priority for improving climate change predictions (Jansson and Hofmockel, 2020). Overall, the convergence of metagenomics, environmental sensing, and ecological modeling represents a new frontier in soil science. This integrative framework enhances our understanding of microbial processes while enabling the development of advanced monitoring systems and evidence-based management strategies. Although previous reviews have examined soil microbial ecology, environmental sensing technologies, metagenomic approaches, or ecological modeling independently, a comprehensive framework integrating these complementary disciplines remains limited. This review addresses this gap by providing a unified perspective that connects advances in soil microbiome research with environmental sensing, metagenomics, ecological modeling, and artificial intelligence-driven analytics. Furthermore, we discuss emerging concepts, including microbiome-informed management and soil digital twins, highlighting their potential to support next-generation predictive soil monitoring and more sustainable soil management strategies under global environmental change.

## 2 Soil as a complex microbial system

Soil represents one of the most complex biological systems in the biosphere, characterized by high spatial heterogeneity and extraordinary microbial diversity, including bacteria, archaea, fungi, protists, and viruses. The interactions among these groups, together with soil physicochemical properties and environmental conditions, contribute to shaping emergent ecosystem functions and processes (Table 1) (Fierer, 2017; Jansson and Hofmockel, 2020). It is estimated that a single gram of soil can harbor thousands of microbial taxa, many of which remain uncultured, highlighting the magnitude of hidden diversity and its functional relevance (Fierer, 2017; Vitorino and Bessa, 2018). Within this context, bacteria dominate in terms of abundance and metabolic plasticity, whereas archaea play key roles in processes such as nitrification and methanogenesis, and fungi contribute significantly to the decomposition of recalcitrant compounds and the formation of mutualistic symbioses with plants (Bardgett and van der Putten, 2014; Jansson and Hofmockel, 2020). Recent evidence from global metagenomic studies has revealed consistent patterns in the functional organization of soil microbial communities, including the worldwide distribution and diversity of diazotrophic groups and their contribution to biological nitrogen fixation across terrestrial ecosystems (Pajares and Bohannan, 2016; Masuda et al., 2024).

**Table 1:** Soil microbial groups, functional traits, and ecosystem services in soil systems.

Microbial group	Representative taxa	Key functional traits/processes	Biogeochemical roles (C, N, P cycling)	Plant interactions	Ecosystem services	References
Bacteria	*Proteobacteria* (*Rhizobium*, *Pseudomonas*), *Actinobacteria* (*Streptomyces*), *Firmicutes* (*Bacillus*)	Rapid growth; metabolic versatility; production of extracellular enzymes (cellulases, proteases); secondary metabolites (antibiotics, phytohormones)	C: decomposition of labile organic matter; N: biological N fixation, nitrification, denitrification; P: solubilization of inorganic phosphate	Plant growth-promoting rhizobacteria (PGPR); phytohormone production (IAA); pathogen suppression; some phytopathogens	Nutrient availability, soil fertility, disease suppression, resilience to disturbance	Fierer (2017), Delgado-Baquerizo et al. (2018), Kuypers et al. (2018)
Archaea	*Thaumarchaeota* (*Nitrososphaera*), *Euryarchaeota*	Adaptation to low nutrient conditions; ammonia monooxygenase activity; chemolithoautotrophy	N: ammonia oxidation (AOA-driven nitrification); C: CO₂ fixation in oligotrophic environments	Indirect effects via N transformations; niche complementarity with bacteria	Stability of N cycling under stress; functioning in extreme or nutrient-poor soils	Prosser and Nicol (2012), Offre et al. (2013), Lu et al. (2015)
Fungi	*Ascomycota* (*Fusarium*, *Penicillium*), *Basidiomycota*, *Glomeromycota* (AMF)	Hyphal growth; ligninolytic and cellulolytic enzymes; formation of mycorrhizal networks	C: decomposition of recalcitrant compounds (lignin, cellulose); P: mobilization via mycorrhizae; N: organic N mineralization	Mycorrhizal symbiosis (enhanced nutrient uptake); increased drought tolerance; pathogenic fungi affect crops	Soil structure formation, carbon sequestration, plant productivity, stress tolerance	Van der Heijden et al. (2008), Treseder and Lennon (2015), Peay et al. (2016)
Protists	*Cercozoa*, *Amoebozoa*, *Ciliophora*	Predation on bacteria and fungi; regulation of microbial populations	C: microbial loop stimulation; N: release of plant-available N via grazing (mineralization)	Indirect promotion of plant growth via microbial turnover and nutrient release	Regulation of microbial community structure; nutrient recycling; ecosystem stability	Geisen et al. (2018), Bonkowski (2004)
Viruses (phages)	Bacteriophages infecting soil bacteria (e.g., Caudovirales)	Host lysis; horizontal gene transfer; auxiliary metabolic genes (AMGs)	C/N: modulation of microbial turnover; reprogramming host metabolism	Indirect effects via control of beneficial/pathogenic bacteria	Microbial diversity regulation; gene flow; ecosystem adaptability	Emerson et al. (2018), Trubl et al. (2018)

This table integrates major soil microbial groups with their functional traits, ecological roles, and contributions to ecosystem services. Soil microorganisms drive key biogeochemical cycles and regulate plant–soil interactions through complex trophic and symbiotic networks, ultimately sustaining soil fertility, productivity, and resilience under environmental change.

From a functional perspective, soil microorganisms are the primary mediators of biogeochemical cycles of carbon (C), nitrogen (N), and phosphorus (P), regulating processes such as decomposition, mineralization, and nutrient transformation, thereby shaping primary productivity and ecosystem dynamics (Delgado-Baquerizo et al., 2016; Basu et al., 2021). Soil acts as a critical carbon reservoir, where microbial activity governs both carbon stabilization and its release as greenhouse gasses, generating key feedback with the climate system (Bertini and Azevedo, 2022; Hao et al., 2025). In this regard, emerging conceptual frameworks suggest that soil microbiomes can be organized into “functional regimes” that condition their responses to environmental change, providing a mechanistic basis for understanding variability in ecosystem processes under global change scenarios (Lee et al., 2025).

The concept of the soil microbiome has evolved toward an integrative framework that encompasses not only taxonomic composition but also functional potential, biotic interactions, and responses to environmental gradients, enabling the interpretation of soil as a complex adaptive system (Hartmann and Six, 2023; Mishra et al., 2023). This perspective recognizes that microbial interactions—including competition, cooperation, and trophic networks—generate emergent properties such as soil fertility, structural stability, and nutrient use efficiency (Sharma et al., 2025). Furthermore, recent studies have shown that microbiome responses to disturbances, such as land-use change or ecological perturbations, are mediated by dynamic feedbacks between community structure and ecosystem function, underscoring the importance of integrating both dimensions in their analysis (Nelson et al., 2024; Lee et al., 2025).

**Figure 2:**
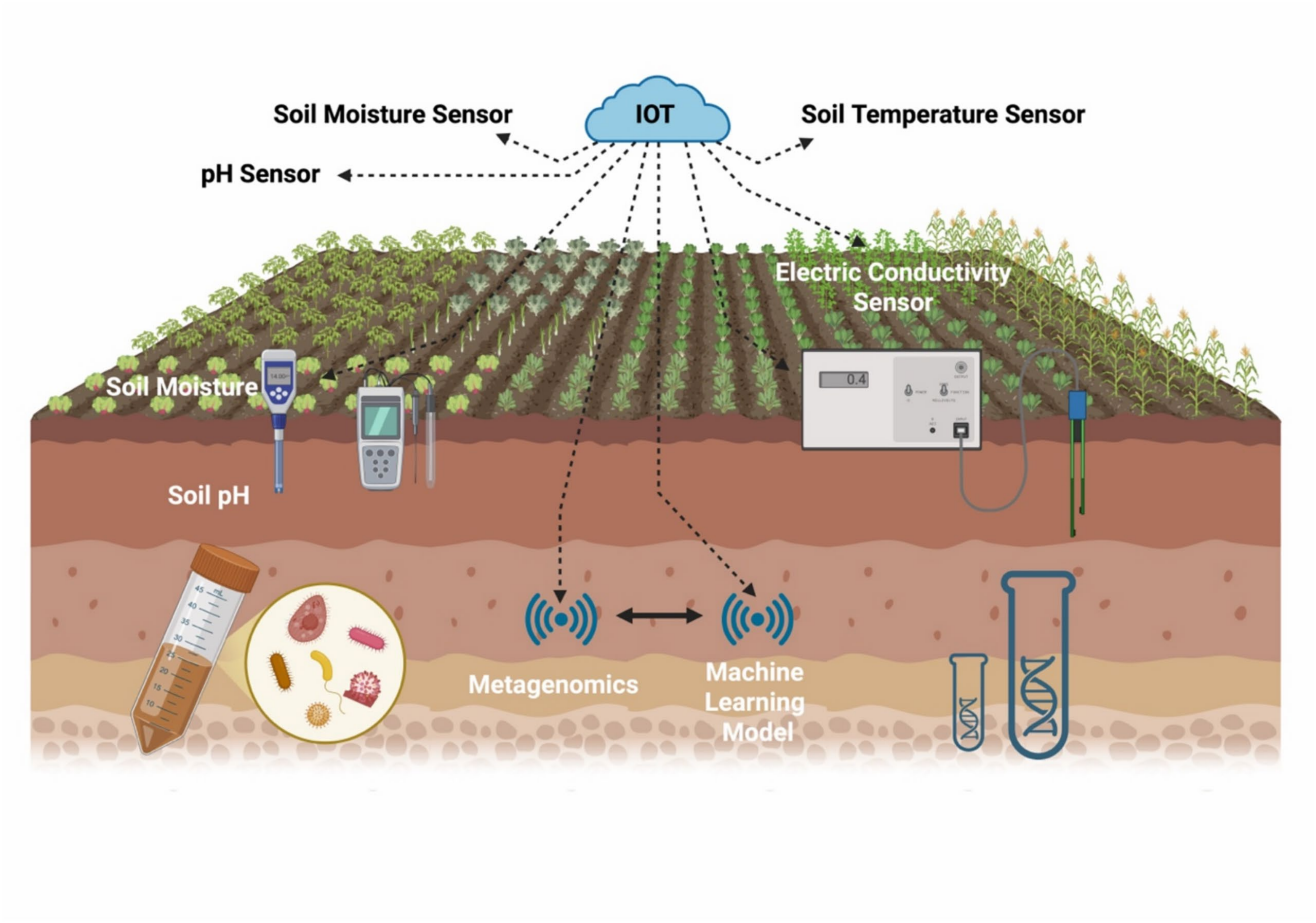
Integrated framework for next-generation soil monitoring. The figure illustrates the integration of *in situ* environmental sensors measuring key soil physicochemical parameters, including moisture, temperature, pH, and electrical conductivity, with Internet of Things (IoT)-based data transmission systems. The framework also incorporates metagenomic sampling and computational data analysis, linking environmental conditions with microbial community structure and function. Together, these components provide high-resolution monitoring of soil systems and can support predictive modeling and data-driven approaches for sustainable soil management.

Resilience and stability emerge as key properties of these complex microbial systems and are closely linked to community diversity and functional redundancy (Philippot et al., 2021). Highly diverse microbial communities tend to exhibit greater resistance to disturbances and faster recovery of ecosystem functions, due to the presence of multiple taxa capable of performing similar roles (Pedrinho et al., 2024). In contrast, biodiversity loss can lead to the simplification of ecological networks and reduced functional stability, increasing system vulnerability to environmental disturbances (Harvey et al., 2017). Recent evidence suggests that microbiome resilience is strongly influenced by its organization into functional regimes and its capacity to reorganize in response to environmental change, with direct implications for the sustainability of terrestrial ecosystems under global change scenarios (Hawkes and Keitt, 2015; Shade, 2023). Overall, these findings highlight the need to approach soil as a complex microbial system whose structure and dynamics are fundamental to the maintenance of critical ecosystem functions (Figure 2).

## 3 Sustainable soil management

Sustainable soil management has emerged as a key approach to address global challenges related to soil degradation, food security, and climate change. This paradigm aims to maintain or enhance soil functionality over the long term through practices that promote biodiversity, structural stability, and resource-use efficiency (Lal, 2012; Telo da Gama, 2023). Compared with highly intensive conventional approaches, sustainable management recognizes soil as a living system in which microbial communities play a central role in regulating critical ecosystem processes (Khaziev, 2011; Raj et al., 2019). Among the most relevant strategies is regenerative agriculture, which promotes practices such as crop rotation, cover cropping, reduced tillage, and the application of organic amendments. These practices have been associated with increases in microbial diversity and activity, may improve soil structure, and can enhance carbon sequestration, although their effects vary depending on soil type, climate, crop system, management context, and the duration of implementation (LaCanne and Lundgren, 2018; Khangura et al., 2023). In particular, reduced tillage minimizes physical soil disturbance, allowing the preservation of microbial niches and fungal networks, which contributes to greater ecological stability (Six et al., 2006; Haddaway et al., 2017). Similarly, cover crops increase organic carbon inputs and stimulate beneficial plant–microorganism interactions (Castellano-Hinojosa and Strauss, 2020).

The use of biofertilizers and plant growth-promoting microorganisms (PGPR) represents another key strategy within sustainable management. These microorganisms can enhance nutrient availability, promote biological nitrogen fixation, and increase plant tolerance to stress conditions (Backer et al., 2018; Chaudhary et al., 2022). In addition, microbial consortia have been proposed as a potentially more robust alternative to single-strain inoculants due to their ability to establish more stable functional networks in soil, although their performance may vary depending on environmental conditions and community composition (Liu et al., 2023; Xu et al., 2025). Reducing the use of synthetic agrochemicals is another fundamental component of sustainable soil management. Excessive application of fertilizers and pesticides has been associated with significant alterations in soil microbiome composition and function, including biodiversity loss and disruption of biogeochemical processes (Geisseler and Scow, 2014; Prashar and Shah, 2016). In contrast, long-term organic or low-input agricultural systems tend to support more diverse and resilient microbial communities compared with highly intensive management practices (Hartmann et al., 2015).

**Figure 3:**
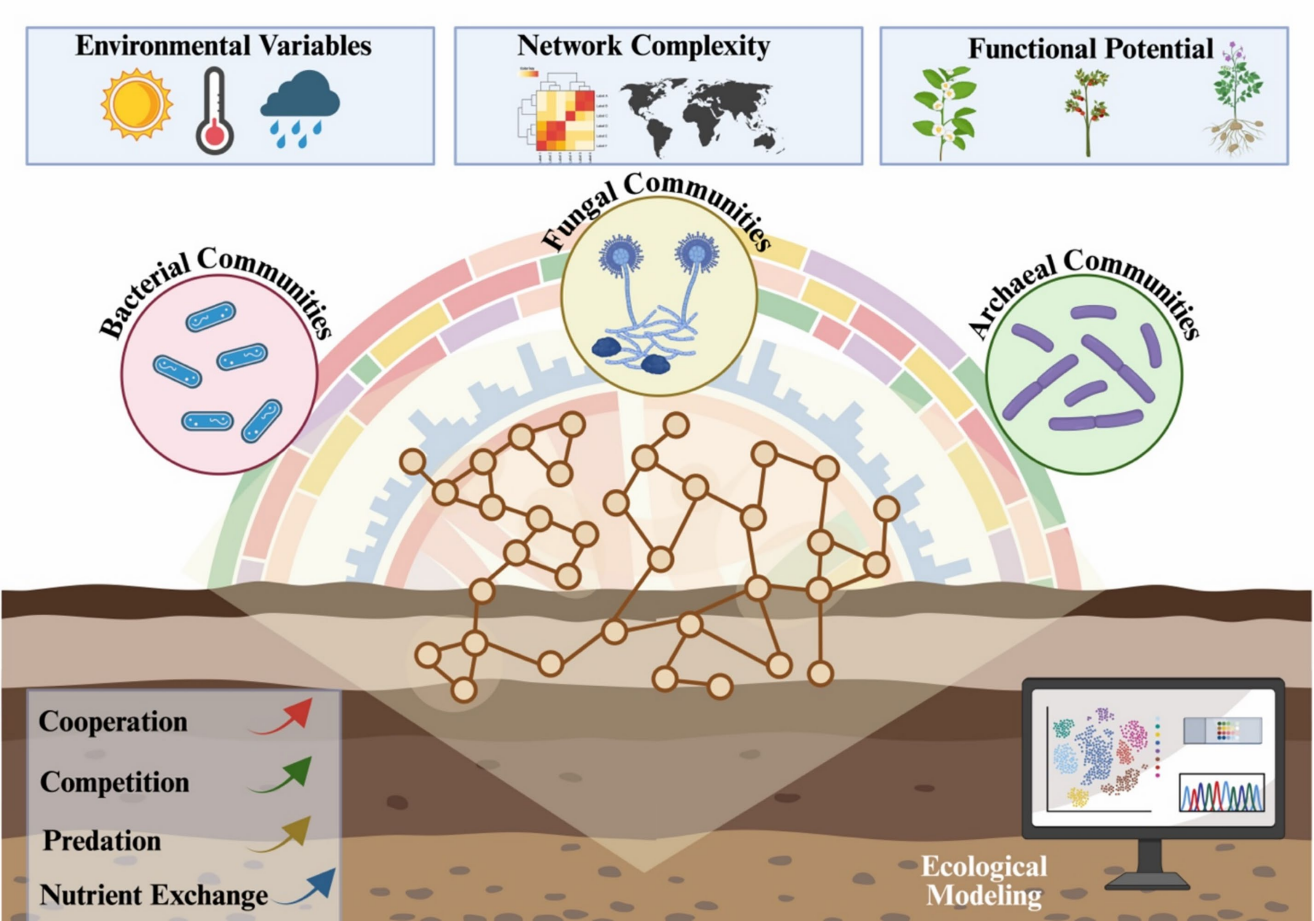
Microbial interaction networks in soil ecological modeling. The figure represents the structure and complexity of soil microbial networks, integrating bacterial, fungal, and archaeal communities as interconnected nodes linked by ecological interactions. These interactions include cooperation, competition, predation, and nutrient exchange, which collectively shape community assembly and ecosystem functioning. Environmental variables such as temperature, moisture, and greenhouse gas dynamics are depicted as external drivers influencing network topology and stability. The integration of network analysis with ecological modeling frameworks is also illustrated, highlighting how these approaches enable the prediction of functional potential, system resilience, and responses to environmental change.

An emerging aspect of sustainable management is the integration of soil microbiome knowledge into the design of agricultural practices. Recent studies suggest that it may be possible to modulate the structure and function of microbial communities through targeted management practices, giving rise to the emerging concept of “microbiome engineering” as a potential strategy to improve agroecosystem productivity and sustainability (Joshi et al., 2025; Toju et al., 2018). However, its application in soil systems remains highly context-dependent and is still under active development. In this context, directed microbiome management may help optimize processes such as nutrient-use efficiency and resistance to pathogens (Ray et al., 2020). Ultimately, sustainable soil management should be understood as an integrative framework that combines agronomic practices, ecological knowledge, and emerging technological tools. The incorporation of microbiome-based approaches, together with technologies such as environmental sensors and ecological modeling, will enable the development of more precise and adaptive strategies to address global change challenges (Keesstra et al., 2024; Barbosa et al., 2025). Collectively, these approaches position the soil microbiome as a central component in the transition toward more resilient, productive, and sustainable agricultural systems (Figure 3).

## 4 Next-generation soil monitoring: sensors and metagenomics

Progress toward sustainable soil management depends largely on the development and integration of tools capable of capturing the biological and environmental complexity of soil systems. In this context, the convergence of environmental sensors, omics technologies—particularly metagenomics—and computational approaches is transforming soil monitoring from a descriptive paradigm into a predictive, data-driven framework (Table 2) (Prosser, 2015; Thompson et al., 2017; Shah et al., 2026; Li et al., 2026). Environmental sensors, including devices for measuring soil moisture, temperature, pH, redox potential, and electrical conductivity, have undergone significant advances in accuracy, miniaturization, and connectivity. The integration of Internet of Things (IoT)-based technologies enables continuous data acquisition at high spatial and temporal resolution, facilitating real-time monitoring of soil conditions (Ray et al., 2017; Yin et al., 2021). These systems allow the detection of dynamic environmental changes, such as fluctuations in soil moisture, temperature, and redox conditions, that directly influence microbial activity and community functioning. Monitoring these variables is essential for understanding key processes, including nutrient mineralization, carbon cycling, and greenhouse gas emissions (Schimel, 2018; Jansson and Hofmockel, 2020; Dutta and Dutta, 2016). However, while sensors provide detailed environmental information, they lack the ability to directly capture the biological dimension of the system. As a result, exclusive reliance on sensor-derived data may overlook transient microbial responses to rapid environmental fluctuations. For example, asynchronous sampling between environmental measurements and microbiome analyses can fail to capture short-lived moisture-driven pulses of microbial activity that strongly influence nutrient transformations and greenhouse gas emissions.

**Table 2:** Comparison of environmental sensors and metagenomic approaches for next-generation soil monitoring.

Tool type	Technology/approach	Variables measured	Spatial–temporal resolution	Strengths	Limitations	Applications	References
Environmental sensors	In situ probes (e.g., moisture, temperature, pH, EC, redox); IoT-enabled systems	Soil moisture, temperature, pH, electrical conductivity, oxygen/redox potential	High temporal (real-time); high spatial (field-scale to plot-scale)	Continuous monitoring; real-time data; low operational cost once deployed; scalable	Limited to physicochemical parameters; no direct biological information; calibration required	Precision agriculture, irrigation management, soil health monitoring, early detection of stress	Ray et al. (2017), Kim et al. (2008), Arshad et al. (2022)
Metagenomics (shotgun)	High-throughput sequencing of environmental DNA	Taxonomic diversity; functional genes; metabolic pathways	Low temporal (snapshot-based); moderate spatial (sampling-dependent)	High-resolution taxonomic and functional profiling; genome reconstruction; discovery of novel taxa	High cost; complex bioinformatics; limited temporal resolution; DNA ≠ activity	Microbiome characterization, functional potential assessment, biodiversity studies	Quince et al. (2017), Nayfach et al. (2021), Almeida et al. (2019a,b)
Amplicon sequencing (16S/ITS)	Targeted sequencing of marker genes	Community composition (bacteria/archaea: 16S; fungi: ITS)	Moderate spatial; low temporal	Cost-effective; standardized; suitable for large-scale comparisons	Limited functional inference; PCR bias; lower taxonomic resolution vs. shotgun	Diversity analysis, community shifts under management or disturbance	Thompson et al. (2017), Walters et al. (2016)
Metatranscriptomics/metaproteomics	RNA sequencing/protein profiling	Gene expression (activity); protein abundance	Low temporal; highly sensitive to sampling conditions	Captures active microbial processes; links function to activity	Expensive; low stability (RNA/proteins); complex workflows	Functional activity profiling; response to environmental change	Prosser (2015), Moran et al. (2013)
Integrated approaches (sensors + omics + AI)	Multi-omics integration with environmental data; machine learning models	Combined physicochemical + biological data	High temporal (sensors) + multi-scale biological resolution	Holistic understanding; predictive capacity; identification of nonlinear relationships	Data integration challenges; computational demands; need for standardization	Predictive soil modeling, decision-support systems, sustainable soil management	Shade et al. (2017), Wilhelm et al. (2022), Basso and Antle (2020)

This table compares the main technological approaches used in next-generation soil monitoring. Environmental sensors provide continuous physicochemical data, while metagenomic and other omics approaches offer insights into microbial diversity and function. Their integration through data science and modeling frameworks enables predictive and data-driven soil management.

In parallel, metagenomics has revolutionized the study of the soil microbiome by enabling the direct analysis of environmental DNA, providing both taxonomic and functional insights into microbial communities (Quince et al., 2017; Nayfach et al., 2021). Shotgun sequencing has enabled the reconstruction of microbial genomes and the characterization of key metabolic pathways, revealing the vast functional diversity of soil systems (Fierer et al., 2012). Furthermore, integration with other omics approaches, such as metatranscriptomics and metaproteomics, provides insights into gene expression patterns and protein production, offering a more dynamic perspective on microbial functional responses beyond genomic potential. However, the interpretation of these data requires caution, and these approaches still face important limitations related to cost, standardization, and data interpretation (Prosser, 2015; Thompson et al., 2017). Similarly, metagenomic analyses performed without synchronized environmental monitoring may provide only a static representation of microbial communities, limiting the ability to link community structure and functional potential to rapidly changing soil conditions.

Despite these advances, one of the major challenges lies in the effective integration of environmental and biological data (Table 2). The disconnect between physicochemical measurements and microbiome information limits the ability to generate mechanistic insights into soil functioning. In this regard, emerging approaches based on data science, artificial intelligence, machine learning, and ecological modeling are enabling the integration of multi-scale and multi-omics datasets to identify patterns and nonlinear relationships among variables (Dutta and Dutta, 2016; Wu and Xie, 2024; Pace et al., 2025; Pace et al., 2026). These computational tools facilitate the development of predictive models capable of assessing microbiome dynamics, soil bio-sustainability, and functional responses to environmental disturbances or management practices. The integration of synchronized environmental sensing and microbiome profiling is therefore essential to improve the temporal resolution and ecological interpretability of soil processes, ultimately supporting more robust predictive frameworks for soil management. Consequently, they support more informed and adaptive soil management strategies and improve our capacity to anticipate changes in soil ecosystem functioning under different environmental scenarios.

## 5 Ecological modeling of soil systems

Understanding soil as a complex biological system has driven the development of ecological modeling approaches capable of integrating multiple levels of biological and environmental organization, including microbial genes, taxa, communities, environmental variables, and ecosystem processes, with the aim of predicting soil dynamics under different environmental and management scenarios (Fierer, 2017; Wieder et al., 2013). In this context, soil microbiome modeling has evolved from descriptive approaches toward predictive frameworks that combine ecological theory, omics data, and advanced computational tools. One of the most relevant approaches is the use of microbial networks, which allow the inference of co-occurrence patterns and potential ecological interactions among taxa. These networks, typically derived from metagenomic or amplicon-based datasets, have revealed that soil microbiomes exhibit highly organized structures, including key nodes (so-called “hub” taxa) that may play disproportionate roles in maintaining system stability (Barberán et al., 2012; Banerjee et al., 2018). The identification of these central taxa has been proposed as a promising approach for understanding and monitoring microbiome organization, as hub taxa may serve as indicators of community structure and function or as candidate targets for further investigation in microbiome management strategies (Agler et al., 2016).

However, network-based approaches are inherently correlative and do not directly infer causal interactions, which limits their predictive capacity under environmental perturbations (Weiss et al., 2016; Srinivasan et al., 2024). In addition, their performance is highly sensitive to compositional bias, sequencing depth, and parameter selection, which can lead to inconsistent network topology across studies (Friedman and Alm, 2012; Kurtz et al., 2015). Methods such as SPIEC-EASI and SparCC have improved robustness by addressing compositionality, yet agreement between inferred interactions remains moderate across algorithms, highlighting a key limitation in reproducibility (Dohlman and Shen, 2019).

Mechanistic modeling frameworks such as MIMICS (Microbial-Mineral Carbon Stabilization model) have been proposed to overcome some of these limitations by explicitly incorporating microbial functional traits and carbon-use strategies into soil organic matter dynamics (Wieder et al., 2015; Dohlman and Shen, 2019). For example, MIMICS has demonstrated improved prediction of soil carbon pools compared to first-order decay models, with reported increases in explanatory power (R^2^ improvements of ~15–30% depending on ecosystem type) and better representation of priming effects under warming scenarios (Wieder et al., 2015; Razavi et al., 2022). However, these models remain constrained by parameter uncertainty, equifinality, and limited transferability across biomes (Shi et al., 2024).

More recently, machine learning approaches such as Random Forest, Gradient Boosting Machines (e.g., XGBoost), and deep neural networks have been increasingly applied to soil microbiome and ecosystem prediction tasks, achieving high predictive performance (often R^2^ > 0.7 for soil carbon, nitrogen, and enzyme activity prediction in benchmark datasets), but at the cost of reduced interpretability and mechanistic insight (Chen and Guestrin, 2016; Padarian et al., 2019). These models excel at capturing nonlinear relationships among environmental and biological variables but may overfit when trained on spatially limited datasets, particularly in heterogeneous soil systems (Smith et al., 2018; Watson, 2022).

Hybrid frameworks that combine mechanistic models with machine learning corrections are emerging as a promising direction, as they integrate process-based interpretability with data-driven predictive power (Reichstein et al., 2019; Razavi et al., 2022). Such approaches have shown improved forecasting accuracy for soil carbon fluxes and microbial activity under climate change scenarios, supporting their potential for next-generation soil system modeling (Ding et al., 2025).

### 5.1 Microbial networks and ecological structure

Microbial networks not only capture co-occurrence relationships but also enable the exploration of emergent properties such as modularity, connectivity, and robustness. Recent studies suggest that higher network complexity may be associated with greater resilience, functional redundancy, and stability in microbial communities under certain environmental conditions. However, these relationships depend on factors such as network architecture, interaction types, modularity, and environmental context. Conversely, the simplification of microbial networks, including that associated with agricultural intensification, has been linked to reductions in soil functional stability and ecosystem multifunctionality (Delgado-Baquerizo et al., 2016; Banerjee et al., 2018). However, it is important to note that correlation-based networks have inherent limitations for inferring causality, which has motivated the development of more robust approaches based on mechanistic models and controlled experimentation (Faust and Raes, 2012). In parallel, mechanistic ecological models are increasingly being used to simulate key soil processes, including organic matter decomposition, carbon dynamics, and nutrient cycling. Models such as MIMICS (Microbial-Mineral Carbon Stabilization) explicitly incorporate microbial biomass and its interactions with the environment, thereby improving predictive capacity compared to traditional models based solely on carbon pools (Wieder et al., 2013; Wieder et al., 2014). These models enable the evaluation of how microbial traits, microbial processes, and microbe–environment interactions can influence ecosystem-scale processes, particularly under climate change scenarios.

### 5.2 Predictive modeling and machine learning

The rise of machine learning has enabled the integration of large volumes of heterogeneous data—including environmental variables, sensor data, and metagenomic profiles—to develop predictive models and improve our capacity to understand and forecast soil responses under different environmental and management conditions. Algorithms such as Random Forest, neural networks, and Bayesian models have been increasingly applied to predict microbiome composition, infer microbial functional potential, and model ecosystem-level processes such as CO₂ and N₂O emissions (Novielli et al., 2024; Barbosa et al., 2025; Olden et al., 2008; Knights et al., 2011; Pace et al., 2025; Pace et al., 2026). These approaches facilitate the identification of complex and nonlinear relationships among environmental, biological, and management variables, improving our understanding of soil ecosystem dynamics. These approaches are particularly powerful in capturing complex and nonlinear relationships, although they often face limitations in biological interpretability.

A recent advance involves the integration of network-based models and machine learning into hybrid frameworks that combine statistical inference with ecological knowledge. These approaches not only improve predictive performance but also enable the generation of hypotheses regarding the underlying mechanisms structuring microbial communities (Willard et al., 2022). In addition, the incorporation of time-series data is facilitating the development of dynamic models capable of capturing microbiome responses to environmental disturbances and management practices.

Finally, the convergence of microbial networks, mechanistic modeling, and machine learning is laying the foundation for integrated predictive soil systems. When combined with real-time data from environmental sensors and omics technologies, these systems support the emergence of a “predictive ecology” framework that not only describes but anticipates soil behavior. Within this context, the identification of key microbial indicators and the modeling of their interactions represent essential tools for designing evidence-based sustainable management strategies (Fierer, 2017; Delgado-Baquerizo et al., 2018).

## 6 Challenges and limitations

Despite significant advances in the study and monitoring of the soil microbiome, several structural challenges still limit the effective implementation of these tools in real-world contexts. One of the main obstacles is the lack of standardization in data generation and analysis, particularly in metagenomic studies. Differences in DNA extraction protocols, sequencing platforms, bioinformatic pipelines, and reference databases hinder cross-study comparisons and the development of coherent global frameworks (Laudadio et al., 2019; Pollock et al., 2018). This methodological heterogeneity reduces reproducibility and constrains the ability to derive robust large-scale inferences.

Another major limitation is the high cost associated with advanced technologies, especially those related to high-throughput sequencing and multi-omics approaches. Although costs have decreased over the past decade, the routine implementation of shotgun metagenomics, metatranscriptomics, or metaproteomics remains restricted in many settings, particularly in resource-limited contexts (Quince et al., 2017; Thompson et al., 2017). Similarly, the deployment of high-resolution sensor networks requires substantial investment in infrastructure, maintenance, and data management, which may limit large-scale adoption.

Biological interpretation of complex datasets represents another critical challenge. Despite the availability of large volumes of data, accurate functional annotation of genes and understanding their ecological relevance remain limited. A significant proportion of genetic material recovered in metagenomic studies corresponds to genes of unknown function, complicating the translation of data into actionable knowledge (Prosser, 2015; Nayfach et al., 2021). Moreover, machine learning models, while highly predictive, often operate as “black boxes,” limiting the mechanistic interpretation of their outputs (Novielli et al., 2024).

Scalability is an additional challenge in the transition from experimental studies to field applications. Results obtained under controlled conditions are not always reproducible in real agricultural systems, where spatial and temporal variability is substantially greater. Furthermore, microbiome-based strategies—such as microbial inoculants or targeted microbiome management—face limitations related to the persistence and stability of introduced microorganisms in soil (Trivedi et al., 2020; French et al., 2021). Finally, integrating data from multiple sources—including sensors, metagenomics, and environmental variables—poses challenges in terms of interoperability, storage, and analysis. The lack of common standards and integrated platforms limits the full exploitation of these approaches (Paraskevas, 2025). Collectively, these challenges highlight the need for more robust methodological and conceptual frameworks to advance toward a truly predictive and operational soil ecology.

## 7 Conclusions and future prospects

The increasing recognition of soil as a complex, living system has reshaped our understanding of its role in sustaining ecosystem functions and agricultural productivity. Throughout this review, we highlight how soil microbiomes act as key regulators of biogeochemical cycles, ecosystem resilience, and plant health, positioning them at the core of sustainable soil management strategies. The convergence of environmental sensing technologies, metagenomics, and ecological modeling is driving a paradigm shift from descriptive soil science toward a predictive and data-driven discipline.

Looking forward, the future of soil management lies in the development of integrative frameworks where the microbiome becomes a central component of decision-making processes. In this context, microbiome-based precision agriculture emerges as a promising approach to optimize crop productivity through the targeted manipulation of microbial communities according to site-specific soil conditions. By combining real-time sensor data, high-resolution microbiome profiling, and predictive models, this strategy has the potential to reduce reliance on chemical inputs while enhancing sustainability and ecosystem functionality.

Another transformative frontier is the development of microbiome engineering approaches, including synthetic microbial consortia and biotechnology-assisted modulation of plant–microbe interactions, supported by advances in biotechnology and synthetic biology. These emerging strategies offer promising opportunities to enhance soil functioning and agricultural sustainability; however, their implementation remains subject to important ecological, regulatory, and biosafety considerations. The design of microbial consortia, the modulation of plant–microbe interactions, and the potential genetic modification of soil microorganisms offer new opportunities to enhance nutrient cycling, stress tolerance, and disease suppression. However, these approaches require a mechanistic understanding of microbial interactions and community assembly processes to ensure ecological stability and minimize unintended consequences.

In parallel, the development of soil digital twins represents a major step toward predictive and adaptive soil management. By integrating real-time environmental data with ecological and computational models, these virtual representations allow the simulation of future scenarios and the optimization of management practices under changing environmental conditions. When coupled with artificial intelligence, digital twins could support more responsive, adaptive, and scenario-based decision-making in soil management, thereby enhancing the capacity to anticipate and respond to environmental and agricultural challenges.

Despite these advances, the successful implementation of microbiome-informed strategies will depend on overcoming key challenges, including data standardization, scalability, and accessibility of advanced technologies. Equally important is the need to strengthen science-based public policies that incorporate microbiome knowledge into soil conservation programs, climate change mitigation strategies, and sustainable agricultural frameworks. Interdisciplinary collaboration among scientists, policymakers, and stakeholders will be essential to translate scientific knowledge into practical solutions. Ultimately, the integration of microbial ecology, technological innovation, and policy frameworks opens the door to a transformative shift in soil management at the global scale. Harnessing the potential of the soil microbiome will be critical to enhancing ecosystem resilience, ensuring food security, and addressing the challenges posed by global environmental change.
